# Supplementary material for: Homologous laminar organization of the mouse and human subiculum
Source: Sci Rep. 2021 Feb 12;11:3729. doi: 10.1038/s41598-021-81362-w (PMC7881248; doi:10.1038/s41598-021-81362-w)
Supplement: Supplementary file 1 — Supplementary Legends [file 41598_2021_81362_MOESM1_ESM.docx]

**Supplementary Table 1.** List of all corresponding gene names, abbreviations, and Entrez Gene ID

**Supplementary Table 2.** Annotation of observed human and mouse gene expression patterns within the four HGEA laminar domains of the SUB, putative interneurons, and glia. ‘X’ indicates positive expression. Annotation as a putative interneuron expression pattern is defined as evenly scattered across pyramidal layer and/or molecular layer of the SUB. Gene expression patterns are annotated based on Allen Institute in situ hybridization database (mouse, <https://mouse.brain-map.org/search/index>; human, <https://human.brain-map.org/ish/search>).

**Supplementary Movie 1.** Anatomical relationship of the human and mouse hippocampus subregions. Yellow = DG, CA3, and CA2, red = CA1, teal = subiculum. In the human, the longitudinal axis is oriented in the anterior/posterior direction whereas the mouse axis is dorsal/ventral. The homologous structure of the hippocampus is similar despite great differences in overall size.

**Supplementary Movie 2.** Human 3D subiculum model. The mouse SUB and human SUB have homologous laminar architecture (SUB_1 = red, SUB_2 = blue, SUB_3= orange, SUB_4 = yellow), but the anatomical position has shifted across evolution. The longitudinal axis of the hippocampus has rotated from dorsal/ventral in the mouse to posterior/anterior in the human. In addition, the anterior pole in human SUB (ventral SUB pole in mouse) has folded back against the longitudinal axis.
